# Supplementary material for: Yiai Fuzheng decoction inhibits triple-negative breast cancer by remodeling the immune microenvironment
Source: Front Immunol. 2025 Sep 30;16:1615631. doi: 10.3389/fimmu.2025.1615631 (PMC12518410; doi:10.3389/fimmu.2025.1615631)
Supplement: Supplementary file 2 [file DataSheet1.zip › Supplementary File 2/Treatment/C_vs_D_vs_H/06_Pathway_Analysis/Pathway_Analysis/Sig_Pathways_ID/Synthesis and degradation of ketone bodies.pdf]

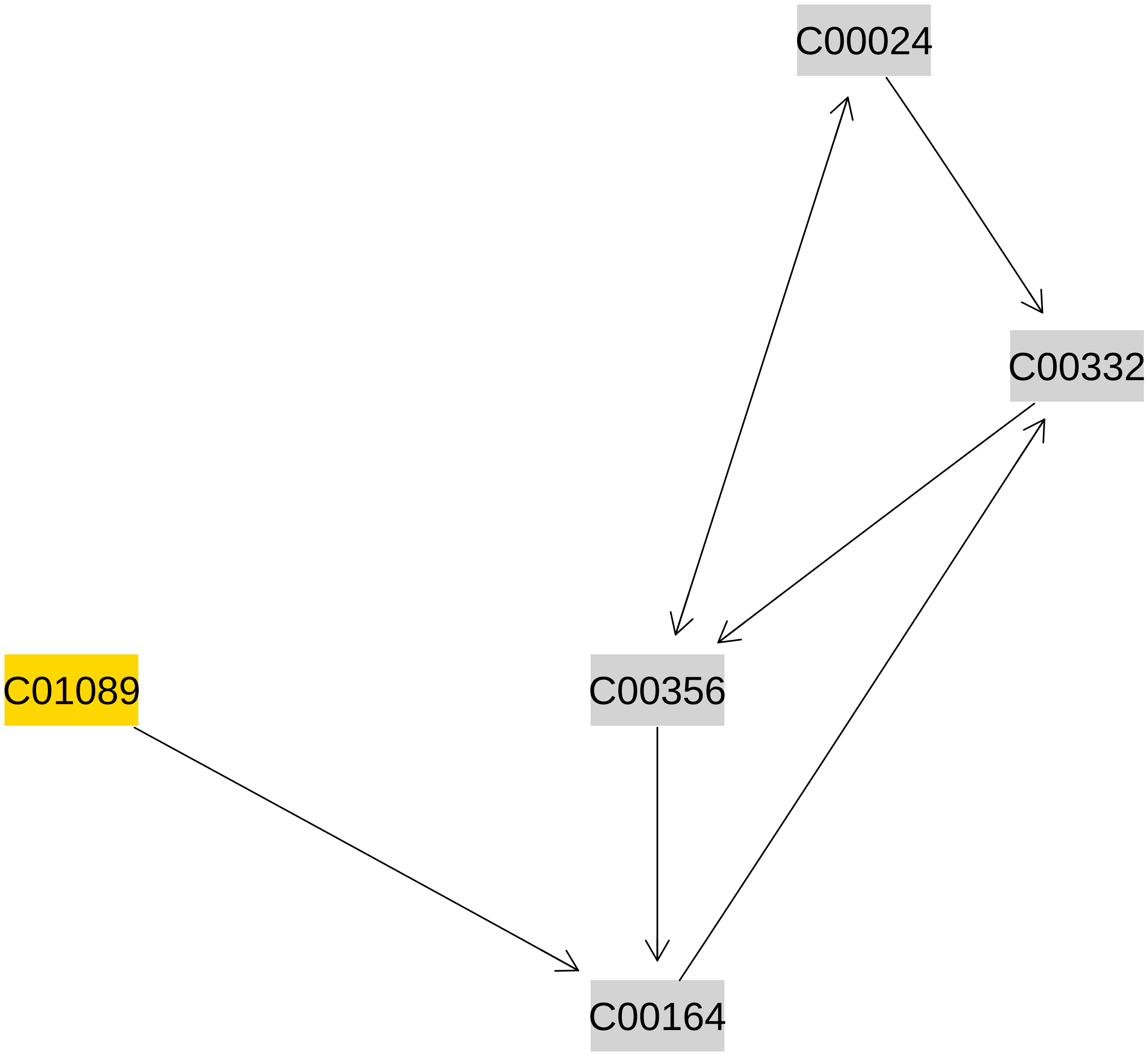
C00024
C00332
C01089
C00356
C00164
